# Identifying clinically relevant drug resistance genes in drug-induced resistant cancer cell lines and post- chemotherapy tissues

**DOI:** 10.18632/oncotarget.5649

**Published:** 2015-10-15

**Authors:** Mengsha Tong, Weicheng Zheng, Xingrong Lu, Lu Ao, Xiangyu Li, Qingzhou Guan, Hao Cai, Mengyao Li, Haidan Yan, You Guo, Pan Chi, Zheng Guo

**Affiliations:** ^1^ Department of Bioinformatics, Key Laboratory of Ministry of Education for Gastrointestinal Cancer, Fujian Medical University, Fuzhou, China; ^2^ Department of Colorectal & Anal Surgery, Affiliated Union Hospital of Fujian Medical University, Fuzhou, China

**Keywords:** drug-induced resistant cancer cell lines, drug treatment response, colorectal cancer, 5-fluorouracil, oxaliplatin

## Abstract

Until recently, few molecular signatures of drug resistance identified in drug-induced resistant cancer cell models can be translated into clinical practice. Here, we defined differentially expressed genes (DEGs) between pre-chemotherapy colorectal cancer (CRC) tissue samples of non-responders and responders for 5-fluorouracil and oxaliplatin-based therapy as clinically relevant drug resistance genes (CRG_5-FU/L-OHP_). Taking CRG_5-FU/L-OHP_ as reference, we evaluated the clinical relevance of several types of genes derived from HCT116 CRC cells with resistance to 5-fluorouracil and oxaliplatin, respectively. The results revealed that DEGs between parental and resistant cells, when both were treated with the corresponding drug for a certain time, were significantly consistent with the CRG_5-FU/L-OHP_ as well as the DEGs between the post-chemotherapy CRC specimens of responders and non-responders. This study suggests a novel strategy to extract clinically relevant drug resistance genes from both drug-induced resistant cell models and post-chemotherapy cancer tissue specimens.

## INTRODUCTION

Differentially expressed genes (DEGs) between parental and drug–induced resistant cells are frequently regarded as drug resistance genes [1–6] and used to identify predictive markers of therapeutic benefit [7–9]. However, findings of such studies can be hardly translated into clinical practice [10–14]. It has been recognized that genes identified from drug–induced resistant cell models may simply represent drug-induced transcriptional changes that may be irrelevant to resistance mechanisms [15]. Therefore, alternative experimental approaches have been proposed.

Stevenson et al. introduced three in vitro gene lists [9]: (i) DEGs between parental and resistant cells [termed basally deregulated (BD) genes]; (ii) DEGs between parental and drug-treated parental cells [inducible in the parental cells (IP) genes], and (iii) DEGs between resistant and drug-treated resistant cells [inducible in the resistant cells (IR) genes]. They considered the pathways significantly enriched with any of the three types of genes as drug resistance pathways. Apparently, both IP and IR genes may mainly represent drug-induced changes, and their relevance to drug resistance is unclear. Allen et al. proposed that the overlap between BD genes and IP genes might represent drug resistance genes [16, 17]. However, because BD and IP genes represent sustained and transient drug-induced changes, respectively, their overlaps may still be irrelevant to drug resistance. Munkácsy et al. proposed that IP genes should be excluded from BD genes [18]. However, some IP genes could be drug resistance genes, and it is difficult to determine which IP genes should be excluded. In contrast to the aforementioned studies, Li et al. proposed that DEGs between a drug-induced resistant cell and its parental cell, both of which have undergone drug treatment for a defined time, might represent targets for therapies aimed at reversing drug resistance [19]. Here, we define this type of DEG as inducible difference (ID) genes, which represent the difference between two cell types in response to drug treatment. Given this diversity of definition for candidate drug resistance genes, it is necessary to evaluate the clinical relevance of various genes identified in cell models.

Another problem is that, in microarray or RNA-sequencing experiments that compare two types of cell lines, usually only two or three technical replicates are generated. Because commonly used statistical methods, such as Significance Analysis of Microarrays (SAM) [20] and variation analysis [21], often have insufficient statistical power when the sample sizes are small [22–24], the FC method is frequently applied to select DEGs in such small-scale cell line experiments [25–27]. However, genes that are highly expressed in both cells can hardly reach large FCs. Moreover, genes with low expression levels in both cell types may reach large FCs owing simply to measurement variations, resulting in false positives [28]. In contrast, the average difference (AD) method can identify genes that are highly expressed in both cells and show large absolute differences, even if the FCs in their expression levels are small [23, 29]. Notably, genes with high expression levels are likely to participate in some biologically conserved pathways, such as metabolism and membrane trafficking [29, 30] Hence, it is necessary to leverage its value in detecting drug resistance genes in small-scale cell line experiments.

In this study, we defined DEGs between pre-chemotherapy clinical tissue samples of responders and non-responders for 5-fluorouracil and oxaliplatin-based therapy as clinically relevant drug resistance genes (CRG_5-FU/L-OHP_). By analyzing the transcriptional profiles of drug-induced resistant cell models, we showed that BD genes mainly reflected drug treatment response and were inconsistent with CRG_5-FU/L-OHP_. In contrast, ID genes, especially when selected according to the AD ranking method, were significantly consistent with CRG_5-FU/L-OHP_. We also found that ID genes were significantly consistent with DEGs between the post-chemotherapy CRC specimens of responders and non-responders, which provided compelling evidence for the use of post-chemotherapy CRC specimens for identifying genes relevant to drug resistance.

## RESULTS

### BD genes are significantly consistent with IP genes

The IP genes were denoted as IP_6_, IP_12_ and IP_24_ for the conditions in which the parental cells underwent drug treatment for 6, 12 and 24 hours, respectively. In the E-MEXP-390 dataset (Table 1), we selected the top-ranked 3000 BD genes and the IP genes for 5-FU with the largest FC values. The consistency scores (the percentage of genes that had the same deregulation directions, see Methods) of BD genes with IP_6_, IP_12_ and IP_24_ were 97.16%, 98.42% and 98.13%, respectively (binomial test, all *P*-values < 1.11E-16, Table 2). When ranking genes by AD, the corresponding consistency scores were 90.92%, 90.96% and 86.85% (binomial test, all *P*-values < 1.11E-16, Table 1). Similarly, significant consistency between BD genes and IP_6_, IP_12_ and IP_24_ genes of L-OHP was observed (binomial test, all *P*-values < 1.11E-16, Table 1). When comparing the top-ranked 1500 BD genes and IP genes, the same results were observed (Supplementary Table 1).

**Table 1:** Datasets analyzed in this study

The cell line data used for identifying BD, IP or ID genes					
Cell line	Dataset	Platform	Drug	Sensitive	Resistant
HCT116(colon)	E-MEXP-390	GPL570	5-FU	3	3
HCT116(colon)	E-MEXP-390	GPL570	L-OHP	3	3
HCT116(colon)	E-MEXP-1691	A-AFFY-101	5-FU	3	3
HCT116(colon)	E-MEXP-1691	A-AFFY-101	SN38	3	3
HCT116(colon)	E-MEXP-171	GPL570	SN38	3	3
EPP85-181P(pancreatic)					
EPG85-257P(gastric)	GSE3926	GPL96	Doxo	1	1
HT29(colon)					
MCF-7(breast)					
DLD1;HT29; LS513;Lovo;(colon)	GSE10405	GPL2006	SN38	1	1

The CRC tissue data used for identifying CRGs					
Stage	Dataset	Platform	Therapeutic regimen	R	NR
IV	GSE19860	GPL570	mFOLFOX6	3	14
IV	GSE28702	GPL570	mFOLFOX6	16	11
IV	E-MEXP-3368	A-AFFY-101	5-FU and L-OHP	4	4
IV	GSE52735	GPL570	5-FU-based	23	14
IV	GSE69657	GPL570	FOLFOX6	13	17

Abbreviations: 5-FU: 5-fluorouracil; L-OHP:Oxaliplatin;SN38: an active metabolite of irinotecan; Doxo: Doxorubicin; R:Responder;NR:Non-responder;

**Table 2:** The consistency scores of the top-ranked 3000 BD and IP_6_, IP_12_, IP_24_ genes detected from HCT1116 cell line

Dataset	Cell line	Drug	Method	IP	Overlapped DEG^a^	Consistent DEG^b^(%)	Binominal *P*-value^c^
E-MEXP-390	HCT116	5-FU	FC	IP_6_	1198	97.16	<1.11E-16
			AD		2026	90.92	<1.11E-16
			FC	IP_12_	1457	98.42	<1.11E-16
			AD		2091	90.96	<1.11E-16
			FC	IP_24_	1229	98.13	<1.11E-16
			AD		2045	86.85	<1.11E-16
E-MEXP-390	HCT116	L-OHP	FC	IP_6_	1898	98.84	<1.11E-16
			AD		2409	98.22	<1.11E-16
			FC	IP_12_	1833	99.95	<1.11E-16
			AD		2298	96.52	<1.11E-16
			FC	IP_24_	1402	99.29	<1.11E-16
			AD		2214	91.64	<1.11E-16
E-MEXP-1691	HCT116	5-FU	FC	IP_24_	1838	98.80	<1.11E-16
			AD		2409	94.69	<1.11E-16
E-MEXP-1691	HCT116	SN38	FC	IP_24_	1934	98.29	<1.11E-16
			AD		2423	95.00	<1.11E-16
E-MEXP-1171	HCT116	SN38	FC	IP_6_	1341	96.20	<1.11E-16
			AD		1974	81.26	<1.11E-16
			FC	IP_12_	1059	88.48	<1.11E-16
			AD		1987	82.94	<1.11E-16
			FC	IP_24_	1079	86.75	<1.11E-16
			AD		2033	86.77	<1.11E-16

Abbreviations: DEG: differentially expressed genes; BD: basally deregulated genes; IP: inducible parental genes; 5-FU: 5-fluorouracil; L-OHP: oxaliplatin; SN38: an active metabolite of irinotecan;

a The number of BD genes overlapped with IP genes;

b The consistency score of BD genes and IP genes;

c The binominal distribution *P*-value.

Subsequent analysis of HCT116 SN-38-resistant cells and doxorubicin-resistant cells from four cancer types (gastric, pancreatic, colon and breast) in the GSE3926 dataset revealed similar results (Supplementary Table 2).

### Clinically relevant drug resistance genes

We defined DEGs between pre-chemotherapy tissue samples of non-responders and responders of CRC patients treated with 5-FU and L-OHP-based therapy as clinically relevant drug resistance genes, denoted as CRG_5-FU/L-OHP_. The GSE19860 and GSE28702 datasets (Table 1), which were both generated by the Affymetrix microarray GPL570 platform, included samples for a total of 25 non-responders and 19 responders of metastatic CRC patients treated with mFOLFOX6 chemotherapy, respectively. We combined the two datasets together to detect 2033 DEGs(FDR < 0.2) using the RankProduct method which is resistant to experimental batch effects [31]. Then, we detected 179 DEGs (FDR < 0.2) between the 4 non-responders and 4 responders of metastatic CRC patients treated with 5-FU and L-OHP in the E-MEXP-3368 dataset. As the mFOLFOX6 regimen also included 5-FU and L-OHP, the overlapped genes of the two lists of DEGs should be CRG_5-FU/L-OHP_ and consistent in deregulation directions under the assumption that the drugs used together in each of the chemotherapy regimens have no or limited antagonistic effects against with each other (Supplementary Methods). In fact, the two lists of DEGs had 82 overlapped genes and the consistency score was 79.27% (binomial test, *P*-value < 5.15E-07). This result suggested that CRG_5-FU/L-OHP_ could be detected robustly in the independent datasets. It also provided evidence for the assumption that the drugs used in combination had no or limited antagonistic effects against with each other. Finally, the 315 DEGs detected with FDR < 0.2 in a dataset and with *P*-value < 0.05 in another dataset were treated as the final CRG_5-FU/L-OHP_ (Supplementary Table 3).

**Figure 1:**
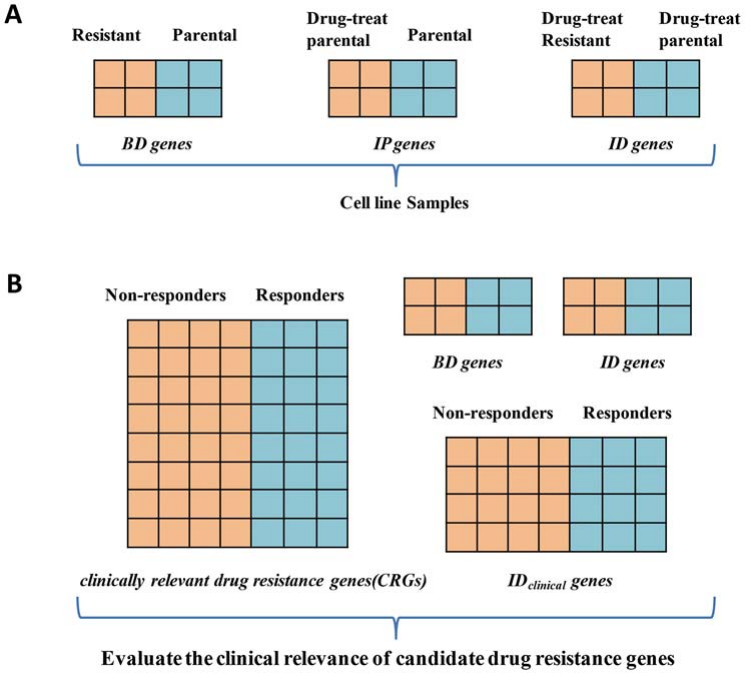
The main idea behind our approach **A.** Three gene lists identified in drug-induced resistant cell models. **B.** Evaluation of clinical relevance of candidate drug resistance gene derived from drug-induced resistant cell models and post-chemotherapy cancer specimens. Abbreviations: BD genes: basally deregulated genes detected between parental cell line and resistant cell line; IP genes: genes detected between parental and drug-treated parental cells; ID genes: genes detected between drug-treat parental cell line and drug-treat resistant cell line; clinically relevant drug resistance genes (CRGs): DEGs between the pre-chemotherapy clinical specimens of responders and non–responders; ID_clinical_ genes: DEGs between the post-chemotherapy clinical specimens of responders and non–responders.

We additionally analyzed the GSE52735 dataset which included samples for 14 non-responders and 23 responders of metastatic CRC patients treated with a combination chemotherapy including fluoropyrimidine (5-FU and capecitabine, an oral prodrug of 5-FU). Using the RankProduct method, we detected 1805 DEGs (FDR < 0.2) between the non-responders and responders and they overlapped with 167 of the 315 CRG_5-FU/L-OHP_. As the three combination chemotherapy regimens shared 5-FU only, the overlapped DEGs should represent CRG_5-FU_ and consistent in deregulation directions in the three gene lists (Supplementary Methods). In fact, the consistency score for the 167 overlapped genes was 78.44% (binomial test, *P*-value = 7.39E-11). This result suggested that CRGs for 5-FU could be detected robustly in independent datasets. The 131 DEGs consistently detected in the three datasets were defined as the final CRG_5-FU_ (Supplementary Table 4). We used CRG_5-FU_ to evaluate the clinical relevance of candidate genes derived from 5-FU resistant cell line models (Figure 1). Due to the lack of other independent datasets for CRC patients with L-OHP-based chemotherapy, we treated the CRG_5-FU/L-OHP_ as the reference to evaluate the clinical relevance of BD genes and ID genes of L-OHP (Supplementary Methods).

### Clinical relevance of BD genes

We defined BD_two_ genes as those found in the overlap between the top-ranked 3000 BD genes of 5-FU and L-OHP, which had the same deregulation directions in the drug-resistant cell lines compared with their corresponding parental cell lines(Supplementary Table 5). The consistency scores between BD_two_ genes and CRG_5-FU/L-OHP_ were as low as 45% for the DEGs ranked by the FC method. The score was 64.44% for the DEGs ranked by the AD method (binomial test, *P*-value = 3.62E-02, Table 3), suggesting significant but weak consistency between BD_two_ genes and CRG_5-FU/L-OHP_. In addition, We evaluated the clinical relevance of BD genes for each single drug. The corresponding consistency scores between the top-ranked 3000 BD genes of 5-FU and CRG_5-FU/L-OHP_ were 51.35% for the FC method and 68.57% for the AD method (Table 3). Similar results were observed when the CRG_5-FU_ were used as the reference to evaluate the clinical relevance of BD genes for 5-FU (Supplementary Table 6). For L-OHP, no significant consistency was observed between BD genes and CRG_5-FU/L-OHP_ when ranking genes by either FC or AD (Table 3). For other CRC L-OHP-resistant cell lines (DLD1, HT29, LS513 and Lovo) in the GSE10405 dataset, no significant consistency was observed between BD genes and CRG_5-FU/L-OHP_ (Table 3).

**Table 3:** The consistency scores of CRG_5-FU/L-OHP_ and the top-ranked 3000 BD genes or ID genes

Dataset	Drug	Cell line	Gene set	Method	Overlapped DEG^a^	Consistent DEG(%)^b^	Binominal *P*-value
E-MEXP-390	5-FU/L-OHP	HCT116	BD_two_	FC	40	45.00	>1.00E-01
				AD	45	64.44	3.62E-02
			ID_two-6_	FC	11	54.55	>1.00E-01
				AD	24	54.17	>1.00E-01
			ID_two-12_	FC	18	55.56	>1.00E-01
				AD	33	57.58	>1.00E-01
			ID_two-24_	FC	10	70.00	1.72E-01
				**AD**	**38**	**84.21**	**1.22E-05**
E-MEXP-390	5-FU	HCT116	BD	FC	74	51.35	>1.00E-01
				**AD**	**70**	**68.57**	**1.27E-03**
			ID_6_	FC	79	55.70	>1.00E-01
				AD	71	52.11	>1.00E-01
			ID_12_	**FC**	**85**	**77.65**	**1.52E-07**
				**AD**	**89**	**74.16**	**2.85E-06**
			ID_24_	FC	79	55.70	>1.00E-01
				**AD**	**79**	**72.15**	**5.13E-05**
E-MEXP-390	L-OHP	HCT116	BD	FC	85	43.53	>1.00E-01
				AD	78	57.69	>1.00E-01
			ID_6_	FC	60	50.00	>1.00E-01
				AD	70	51.43	>1.00E-01
			ID_12_	FC	92	28.26	>1.00E-01
				AD	82	35.37	>1.00E-01
			ID_24_	**FC**	**71**	**74.65**	**1.94E-05**
				**AD**	**71**	**83.10**	**6.74E-09**
GSE10405	L-OHP	DLD1	BD	FC	20	70.00	>1.00E-01
				AD	21	57.14	>1.00E-01
		HT29		FC	26	42.31	>1.00E-01
				AD	16	43.75	>1.00E-01
		LS513		FC	18	55.56	>1.00E-01
				AD	21	52.38	>1.00E-01
		Lovo		FC	23	43.48	>1.00E-01
				AD	15	53.33	>1.00E-01

Abbreviations: ID: inducible difference genes;

a The number of candidate drug resistance genes overlapped with CRG_5-FU/L-OHP_;

b The consistency score of candidate drug resistance genes and CRG_5-FU/L-OHP_.

Similar results were observed when analyzing the top-ranked 1500 BD_two_ and BD genes ranked by FC or AD (Supplementary Table 7). These results suggest that the clinical relevance of BD genes is poor.

### Clinical relevance of ID genes

We defined ID_two_ genes as the overlap of the top-ranked 3000 ID genes of 5-FU and L-OHP, which had the same deregulation directions in the drug-resistant cell lines treated with 5-FU or L-OHP compared with their corresponding parental cell lines also treated with 5-FU or L-OHP (Supplementary Table 5). The ID_two_ genes were further denoted as ID_two_-_6_, ID_two_-_12_ and ID_two_-_24_ for the conditions where cells underwent drug treatment for 6, 12, and 24 hours, respectively. We then evaluated the clinical relevance of these genes. No significant consistency was observed for ID_two_-_6_ and ID_two_-_12_ genes when ranking genes either by FC or AD (Table 3). For ID_two_-_24_ genes, however, the consistency score was as high as 84.21% when ranking genes by AD (binomial test, *P*-value=1.22E-05). The score was also as high as 70% when ranking genes by FC, although it did not reach significance. Similar results were observed when analyzing the top-ranked 1500 ID_two_ genes (Supplementary Table 7).

We further evaluated the clinical relevance of the top-ranked 3000 ID genes of 5-FU. When ranking genes by FC, only ID_12_ genes were significantly consistent with CRG_5-FU/L-OHP_, with a corresponding consistency score of 77.65% (binomial test, *P*-value = 1.52E-07, Table 3). When ranking genes by AD, however, significant consistency was observed for both ID_12_ and ID_24_ genes and the corresponding consistency scores were 74.16% (binomial test, *P*-value = 2.85E-06) and 72.15% (binomial test, *P*-value = 5.13E-05, Table 3), respectively. Similar results were observed when the CRG_5-FU_ were used as the reference to evaluate the clinical relevance of ID genes for 5-FU (Supplementary Table 6). With regard to L-OHP, no significant consistency was observed for either ID_6_ or ID_12_ genes when ranking genes by either FC or AD (Table 3). For ID_24_ genes, however, the corresponding consistency scores were 74.65% when ranked by FC (binomial test, *P*-value = 1.94E-05) and 83.10% by AD (binomial test, *P*-value = 6.74E-09, Table 3). Similar results were observed when analyzing the top-ranked 1500 ID genes (Supplementary Table 7).

Additionally, we applied two-way analysis of variance to identify ID genes. The numbers of ID genes for L-OHP and 5-FU were 269 and 361 (*P*-value < 5.00E-02), respectively, and they overlapped with only 6 and 3 of CRG_5-FU/L-OHP_, respectively, due to the limited efficiency of variance estimation [22].

**Figure 2:**
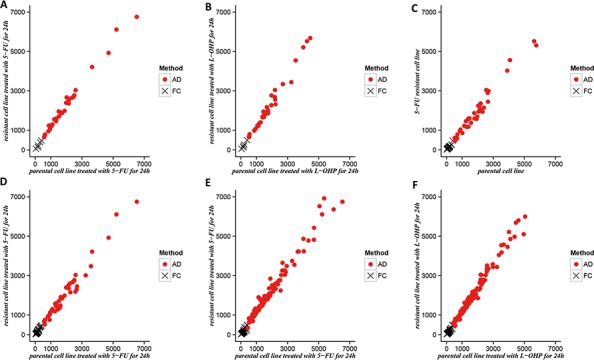
The distributions of DEGs exclusively detected by FC or AD The average expression levels of DEGs exclusively detected by FC or AD were plotted. **A–B.** ID_two_-_24_ genes compared with CRG_5-FU/L-OHP_ in parental cell line treated with 5-FU(or L-OHP) for 24 h and resistant cell line treated with 5-FU(or L-OHP) for 24 h; **C.** BD genes of 5-FU compared with CRG_5-FU/L-OHP_ in parental cell line and 5-FU resistant cell line; **D.** ID_24_ genes of 5-FU compared with CRG_5-FU/L-OHP_ in parental cell line treated with 5-FU for 24 h and resistant cell line treated with 5-FU for 24 h; **E–F.** ID_two_-_24_ genes compared with ID_clinical_ genes in parental cell line treated with 5-FU(or L-OHP) for 24 h and resistant cell line treated with 5-FU(or L-OHP) for 24 h;

We found that the ID_two_-_24_ genes, BD genes of 5-FU and ID_24_ genes of 5-FU detected by the AD method were more significantly consistent with CRG_5-FU/L-OHP_ compared with the FC method (Table 3). With regard to the ID_two_-_24_ genes, there were 35 genes detected by AD but not FC. The average expression levels of these genes in parental cells treated with 5-FU for 24 hours and 5-FU-resistant cells treated with 5-FU for 24 hours were 1992.72 and 2322.72 (Figure 2A, Supplementary Table 8). The consistency score of these genes with CRG_5-FU/L-OHP_ was 82.86% (binomial test, *P*-value = 5.84E-05, Supplementary Table 8). By contrast, 7 genes detected by FC but not AD tended to have low expression levels and the corresponding average expression levels were 212.93 and 238.03 (Figure 2A, Supplementary Table 8). The corresponding consistency score was 57.14% (Supplementary Table 8). A similar result was also observed in L-OHP-resistant cells (Figure 2B, Supplementary Table 4). Subsequent analysis of BD genes of 5-FU and ID_24_ genes of 5-FU revealed similar results (Figure 2C–2D, Supplementary Table 8). These results demonstrate that AD is biased toward the identification of genes expressing at higher levels, whereas FC is biased at lower levels. Genes with low expression levels in both cell lines may reach large FCs simply due to measurement variations that create false positives [32].

**Figure 3:**
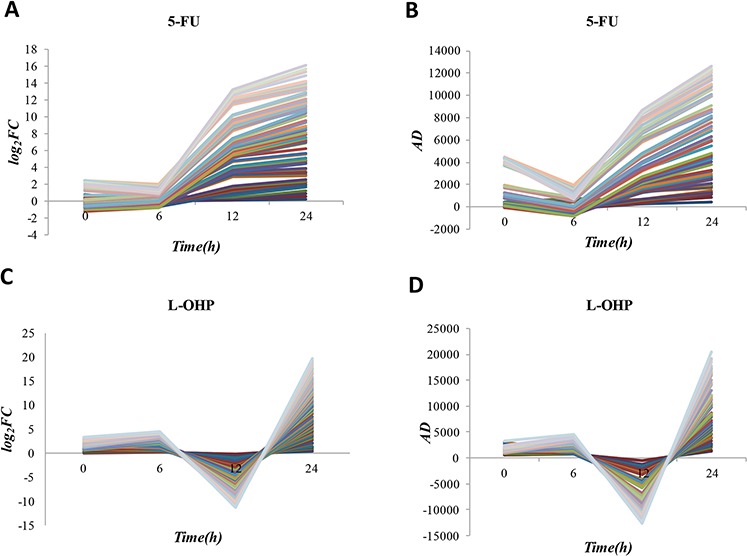
The log2 FC values and AD values of 70 genes of 5-FU resistance and 65 genes of L-OHP resistance **A–B.** The log_2_ FC values and AD values of 70 genes of 5-FU resistance in parental cell line treated with 5-FU for 24 h and resistant cell line treated with 5-FU for 24 h; **C–D.** The log_2_ FC values and AD values of 65 genes of L-OHP resistance in parental cell line treated with L-OHP for 24 h and resistant cell line treated with L-OHP for 24h.

It is worth noting that ID_two_-_24_ and ID_24_ genes of both 5-FU and L-OHP had significant consistency with CRG_5-FU/L-OHP_ while no significant consistency was observed in ID_two_-_6_ and ID_6_ genes (Table 2). We combined ID_24_ genes detected by FC or AD method, which were significantly consistent with CRG_5-FU/L-OHP_, resulting in 70 genes of 5-FU resistance and 65 genes of L-OHP resistance (Supplementary Table 9). The log_2_ FC values and AD values of these genes are shown in Figure 3A–3D. We found that resistant genes of both 5-FU and L-OHP tended to change abruptly before the 24-hour time point. This result indicates that transient changes in expression levels might be unstable when the drug treatment time is short. It has been reported that many of the genes obtained above correlate with drug resistance, as exemplified in Supplementary Table 10 for the top 20 ID_24_ genes ranked by AD method for each of the two drugs. TYMS is target of 5-FU and its overexpression can induce 5-FU resistance [33]. UNG can initiate base excision repair and its overexpression may stimulate the development of L-OHP resistance [34]. Up-regulation of PSAT1 stimulates cell growth and increases chemoresistance of colon cancer cells to L-OHP [35]. TTK, MCM2, CLDN7 and TSPAN13 promote tumor cell proliferation and their overexpression could stimulate drug resistance [36–39].

### Pathway analysis of ID_24_ genes

Functional enrichment analysis showed that the top 3000 ID_24_ genes of 5-FU separately ranked by FC and AD were enriched in 6 and 22 pathways, respectively (FDR < 0.1, Supplementary Table 11). With regard to L-OHP, the top 3000 ID_24_ genes separately ranked by FC and AD were enriched in 14 and 31 pathways, respectively (FDR <0.1, Supplementary Table 11). It has been reported that many of the pathways enriched with ID_24_ genes could mediate drug resistance of the corresponding drug, as described in the Supplementary Table 11. Genes detected by the AD method but not FC were significantly enriched mostly in more conserved pathways with important biological significance, including glycolysis/gluconeogenesis, citrate cycle (TCA cycle), fatty acid degradation and glutathione metabolism. It has been found that targeting metabolic enzymes in the glycolytic pathway, citric acid cycle and fatty acid synthesis could enhance the efficacy of common therapeutic agents and overcome resistance to chemotherapy [40]. Elevation of glutathione metabolism pathway involved in the deactivation of anticancer agents [41]. Several inhibitors which have been reported to target the corresponding pathways were listed in the Supplementary Table 12.

### Identification of ID genes based on post-chemotherapy CRC specimens

We denoted ID_clinical_ genes as DEGs between the post-chemotherapy CRC specimens of responders and non–responders, which are similar to ID genes that represent the difference between two cell types in response to drug treatment. Using the RankProduct method, we detected 1725 ID_clinical_ genes (FDR < 0.1), with the consistency score between ID_clinical_ genes and CRG_5-FU/L-OHP_ (CRG_5-FU_) as high as 83.85% (88.46%) (binomial test, *P*-value < 1.11E-16, Supplementary Table 13). Furthermore, the consistency score between ID_clinical_ genes and the top-ranked 3000 ID_two_-_24_ detected by the AD method was 73.57% (binomial test, *P*-value < 1.09E-08, Supplementary Table 13). Both ID_two_-_24_ and ID_24_ genes were substantially more consistent with ID_clinical_ genes than ID_two_-_6_, ID_two_-_12,_ ID_6_, and ID_12_ (Supplementary Table 13). Similar results were observed when analyzing the top-ranked 1500 ID genes ranked by FC or AD (Supplementary Table 13). In addition, the ID_two_-_24_ genes detected by the AD method were also more significantly consistent with ID_clinical_ genes compared with the FC method (Supplementary Table 8, Figure 2E–2F).

## DISCUSSION

Current cancer therapeutics are generally dosed in combination [42, 43]. This makes it difficult to directly study drug resistance mechanisms for any single drug in clinical cohorts. Thus, using cell models would be the only practical choice for identifying resistant signatures for individual drugs [7, 44–46] although the clinical relevance of cancer cell models has been continuously questioned [10–14]. Our results demonstrated that, rather than BD genes, ID genes which represented the difference between parental and resistant cells in response to drug treatment would be more likely to be involved in drug resistance. Moreover, our analysis supports that samples taken after neoadjuvant chemotherapy can be used to ascertain functional drug resistance signatures [47, 48].

One caveat of our analysis for identifying ID genes is that expression profiles of drug-treated cells were only measured at 6, 12, and 24 hours, which might be insufficient to investigate the characteristics of sustained responses [49, 50]. It would be possible that the difference between two types of cell in sustained response to drug treatment might be more likely relevant with drug resistance. Hence, it is necessary to design experiments with prolonged time of drug treatment and to further characterize the dynamic transcriptome change. Another problem is that many factors such as the choice of a parent cell line, drug dose and treatment interval are associated with the drug resistance level of the drug-induced resistant cell [51]. A reasonable assumption would be that using cell models with higher level of drug resistance might have larger chance to find drug resistance genes, which deserves our future study. The third problem is that the CRGs identified from pre-chemotherapy specimens represent inherent resistance genes, whereas the process of inducing a drug sensitive cell to become a drug resistant cell by drug treatment might mimic the process of acquiring drug resistance for clinical patients during chemotherapy. However, it has been suggested that there might be no obvious boundaries between inherent drug resistance genes and acquired drug resistance genes [52, 53]. In fact, the significant consistency between the ID genes and the corresponding CRGs could be regarded as evidence supporting the notion that the two type genes might be largely consistent [52, 53].

In summary, this pilot study on CRC suggests a novel experimental analysis strategy to extract clinically relevant drug-resistance signatures from drug-induced resistant cell models. It also suggests that tumor tissue samples taken at definitive surgery after chemotherapy could be useful for identifying drug-resistance signatures.

## MATERIALS AND METHODS

### Data acquisition and processing

The transcriptional profiles of 30 post-chemotherapy CRC specimens were submitted to Gene Expression Omnibus (GEO) under accession number GSE69657. All patients underwent neoadjuvant FOLFOX4 chemotherapy, and there were 13 responders and 17 non-responders according to the Response Evaluation Criteria in Solid Tumors (RECIST) [54]. The detailed experimental protocols were described in a previous study [55]. All other datasets analyzed in this study were downloaded from GEO and ArrayExpress (Table 1). The datasets generated from the Affymetrix microarray platform were pre-processed using the robust microarray average (RMA) algorithm and the other datasets generated from the Illumina and Agilent microarray platform were log2-transformed and quantile normalized. Normalization of GSE10405 was performed with Lowess and Dye Swap Sim Fix Filter methods [56]. Each probe-set ID was mapped to its Entrez gene ID. If multiple probe-sets were mapped to the same gene, the expression value for the gene was defined as the arithmetic mean of the values of the multiple probe-sets.

### Reproducibility evaluation of DEGs from independent datasets

For DEGs from two independent datasets, sharing k DEGs, of which s genes had the same deregulation directions (both up-regulated or down-regulated in the two gene lists), the consistency score was calculated as s/k. The probability of observing at least s of k DEGs with the same deregulation directions by chance can be evaluated using the cumulative binomial distribution model as follows:

$$p=1-\sum_{i=0}^{s-1}\left(\begin{array}{c} k\\ i \end{array}\right){\left(p_{e}\right)}^{i}{\left(1-p_{e}\right)}^{k-i}$$ (1)

where *p_e_* is the probability of one gene having the same deregulation directions in two gene lists by random chance (here, *p_e_*=0.5).

### Selection of clinically relevant drug resistance genes

We have made a strict mathematical derivation to prove that if two different regimens share one or several drugs, then the overlaps of CRGs for the two different regimens should be the CRGs for the shared drug(s) under the assumption that the drugs used in combination had no (or limited) antagonistic effects (Supplementary Methods). The RankProduct method [31], which is resistant to batch effects, was applied to identify DEGs between responders and non–responders. Using 52 samples collected from three independent datasets (Table 1), we selected and evaluated the reproducibility of the CRG_5-FU/L-OHP_. The GSE52735 dataset including 37 samples was used to select the CRG_5-FU_. The *p*-values were adjusted using the Benjamini and Hochberg procedure [57].

### Selection of DEGs from cell lines

The non-log-transformed average expression values of gene *i* in the drug-resistant sample and parental sample were denoted as $X_{i}^{A}$ and $X_{i}^{B}$, respectively. Then, the FC for each gene *i* between the two samples was calculated as follows:

$${\text{FC}}_{i}=\frac{X_{i}^{A}}{X_{i}^{B}}$$ (2)

The AD for each gene *i* between the two samples was calculated as follows:

$${\text{AD}}_{i}=X_{i}^{A}-X_{i}^{B}$$ (3)

All genes were sorted in descending order according to FC or AD. If the value of *FC_i_* was larger (or smaller) than one, then gene *i* was defined as up-regulated (or down-regulated) in resistant samples. Similarly, if the value of *AD_i_* was larger (or smaller) than zero, gene *i* was defined as up-regulated (or down-regulated) in resistant samples.

### Pathway enrichment analysis

Functional enrichment analysis was performed based on the Kyoto Encyclopedia of Genes and Genomes [58]. The hypergeometric distribution model was used to identify biological pathways that were significantly enriched with DEGs.

## SUPPLEMENTARY DATA TABLES


