# Supplementary material for: Identifying clinically relevant drug resistance genes in drug-induced resistant cancer cell lines and post- chemotherapy tissues
Source: Oncotarget. 2015 Oct 15;6(38):41216–27. doi: 10.18632/oncotarget.5649 (PMC4747401; doi:10.18632/oncotarget.5649)
Supplement: Supplementary file 1 [file oncotarget-06-41216-s001.pdf]

SUPPLEMENTARY DATA

Two drugs used together is pharmacodynamically synergistic, additive or antagonistic if the therapeutic effect is greater than, equal to, or less than the summed effects of the partner drugs [47]. Drug combinations may also produce pharmacokinetically potentiative or reductive effects such that the therapeutic activity of one drug is enhanced or reduced by another drug [47]. Another type of drug combination is a coalistic combination, in which all of the drugs involved are inactive individually but are active in combination [48]. In this study, we only consider three pharmacodynamical types of drug combinations: synergistic, additive and antagonistic effects.

We define the DEGs between the non-responders and responders of patients treated with drug A and B as CRGs for the combination chemotherapy, denoted as $CRG_{A/B}$. The DEGs between the non-responders and responders of patients treated with drug A or B alone were defined as CRGs for drug A or CRGs for drug B, denoted as CRG_A or CRG_B, respectively. If CRG_A and CRG_B have overlaps, then we define each of the overlapped genes as a synergistic or antagonistic gene if it has the same or opposite deregulation directions (up-regulation or down-regulation) in the non-responders compared with the responders) in CRG_A and CRG_B. Then, we can prove the following conclusions:

1. If a CRG_A (CRG_B) is not a CRG_B (CRG_A), then it is statistically expected to be detected as a $CRG_{A/B}$ with the same deregulation direction in non-responders compared with responders.
2. For a synergistic gene included in both CRG_A and CRG_B, it is expected to be included in $CRG_{A/B}$ with the same deregulation direction.
3. For an antagonistic gene, it may or may not be detected as a $CRG_{A/B}$. If it is also detected as a $CRG_{A/B}$, then its deregulation direction could be inconsistent with CRG_A or CRG_B.
4. If the frequency of antagonistic genes in CRG_A or CRG_B is small, then the consistence score between the CRG_A or CRG_B and $CRG_{A/B}$ should be significant.
5. The same conclusions can be proven for combination chemotherapy with more than two drugs.

Under the same assumption that the frequency of antagonistic genes is small, we can prove the following conclusion:

6. If two different regimens share one or several drugs, then the overlaps of CRGs for the two different regimens should be the CRGs for the shared drug(s).

Proof. We assume that the non-responders are resistant to all the drugs used together (subtype0) and the responders have three subtypes: sensitive to drug B only(subtype1), sensitive to drug A only(subtype2), and sensitive to both A and B(subtype3). For any of CRG_A, denoted as g_A, we suppose that expectation of its expression value in subtype i is μ_{gAi} (i = 0,1,2,3). The frequency of subtypes in the non-responder and responder groups for combination chemotherapy is f_i(i=0,1,2,3, $f_1+f_2+f_3=1$).

The four kinds of sample subtypes were displayed in the following table:

	drug A	drug B	μ_{gAi}	f_i
non-responder	1	1	μ_{gA0}	f_0
	1	0	μ_{gA1}	f_1
responder	0	1	μ_{gA2}	f_2
	0	0	μ_{gA3}	f_3

Note: 1(0): resistance(sensitive) to one drug;

μ_{gAi}: the expectation of expression value of g_A in the non-responder and responder groups(i=0,1,2,3);

f_i: the frequency of subtypes in the non-responder and responder groups(i=0,1,2,3).

According the table above, $\mu_{gA0}-\mu_{gA2}$ and $\mu_{gA1}-\mu_{gA3}$ both represent the difference of g_A in the non-responders compared with the responders for drug A, we define

$$\Delta_A(g_A)=\mu_{gA0}-\mu_{gA2}=\mu_{gA1}-\mu_{gA3}$$

Similarly,

$$\Delta_B(g_A)=\mu_{gA0}-\mu_{gA1}=\mu_{gA2}-\mu_{gA3}$$

We further define $\Delta_{A/B}(g_A)$ as the difference of g_A in the in the non-responders compared with the responders for combination chemotherapy with drug A and drug B, then,

$$\because \mu_{gA0}-\mu_{gA1}=\mu_{gA2}-\mu_{gA3}$$
$$\therefore \mu_{gA3}=\mu_{gA2}+\mu_{gA1}-\mu_{gA0}$$
$$\because f_1+f_2+f_3=1$$
$$\begin{aligned}\therefore \Delta_{A/B}(g_A)&=\mu_{gA0}-(f_1\mu_{gA1}+f_2\mu_{gA2}+f_3\mu_{gA3})\\ &=\mu_{gA0}(f_1+f_2+f_3)-(f_1\mu_{gA1}+f_2\mu_{gA2}+f_3\mu_{gA3})\\ &=f_1(\mu_{gA0}-\mu_{gA1})+f_2(\mu_{gA0}-\mu_{gA2})+f_3(\mu_{gA0}-\mu_{gA3})\\ &=f_1\Delta_B(g_A)+f_2\Delta_A(g_A)+f_3(\mu_{gA0}-\mu_{gA2}-\mu_{gA1}+\mu_{gA0})\\ &=f_1\Delta_B(g_A)+f_2\Delta_A(g_A)+f_3(\Delta_B(g_A)+\Delta_A(g_A))\\ &=(1-f_1)\Delta_A(g_A)+(1-f_2)\Delta_B(g_A)\end{aligned}$$

For $\Delta_{A/B}(g_A)=(1-f_1)\Delta_A(g_A)+(1-f_2)\Delta_B(g_A) \quad (0\le f_1\le 1, 0\le f_2\le 1)$

we suppose $\Delta_A(g_A)$ > 0, which means g_A is up-regulated in the non-responders compared with the responders for drug A.

1. if $g_A \notin (CRG_A \cap CRG_B)$ then $\Delta_B(g_A)$ = 0,which means g_A has no difference in the non-responders

compared with the responders for drug B and the two drugs act independently to each other. Thus $\Delta_{A/B}(g_A) = (1 - f_1)\Delta_A(g_A) \geq 0$;

2. If $g_A \in (CRG_A \cap CRG_B)$ and $\Delta_B(g_A) > 0$,which means g_A is a synergistic gene, then $\Delta_{A/B}(g_A) = (1 - f_1)\Delta_A(g_A) + (1 - f_2)\Delta_B(g_A) \geq 0$;
3. If $g_A \in (CRG_A \cap CRG_B)$ and $\Delta_B(g_A) < 0$,which means g_A is an antagonistic gene,

Only when $\frac{-\Delta_A(g_A)}{\Delta_B(g_A)} < \frac{1-f_2}{1-f_1} = \frac{1-f_2}{f_2+f_3} \leq \frac{1-f_2}{f_2}$ then, $\Delta_{A/B}(g_A) < 0$

$$\because \Delta_A(g_A) > 0, \Delta_B(g_A) < 0$$

$$\therefore \frac{-\Delta_A(g_A)}{\Delta_B(g_A)} > 0$$

Only when f_2 which represents the sample size of responders who are sensitive to drug A is small enough, then $\Delta_{A/B}(g_A) < 0$. In fact, accumulated empirical clinical experience showed that cytotoxic drugs given in combination was to achieve additive or synergistic effects [49].

Similarly, the same conclusions can be proven when g_A down-regulated in the non-responders compared with the responders for drug A. The mathematical derivations above can be summarized to the following derivations more concisely:

G: the whole genes set

$\Delta_X(g)$: the expection difference of gene g between the sensitive and resistant samples of a drug X.

$$CRG_A = \{g | g \in G \wedge \Delta_A(g) \neq 0\}$$
$$CRG_B = \{g | g \in G \wedge \Delta_B(g) \neq 0\}$$

Let

$$CRG_{A/B} = CRG_A \cup CRG_B$$
$$= (CRG_A - CRG_B) \cup (CRG_B - CRG_A) \cup (CRG_A \cap CRG_B)$$
$$CRG = \{g | g \in G \wedge \Delta_g \neq 0\}$$

no overlap

$$\because \forall g \in CRG_A \rightarrow \Delta_A(g) \neq 0$$
$$\therefore \forall g \in (CRG_A - CRG_B) \rightarrow \Delta_A(g) \neq 0$$

Similarly,

$$\forall g \in (CRG_B - CRG_A) \rightarrow \Delta_B(g) \neq 0$$
$$\because \forall g \in (CRG_A - CRG_B) \rightarrow \Delta_g = (1 - f_I)\Delta_A(g) \propto \Delta_A(g) \neq 0 \; (0 \leq f_I \leq 1)$$
$$\therefore CRG_A - CRG_B \subset CRG$$

Similarly,

$$CRG_B - CRG_A \subset CRG$$

synergistic gene

$$\exists g \in (CRG_A \cap CRG_B) \wedge (\Delta_A(g)\,\Delta_B(g) > 0) \rightarrow \Delta_g = (1 - f_I)\Delta_A(g) + (1 - f_2)\Delta_B(g) \neq 0$$
$$\{g | g \in (CRG_A \cap CRG_B) \wedge (\Delta_A(g)\,\Delta_B(g) > 0\} \subset CRG$$

antagonistic gene

1, comfusing direction

$$for\ \{g | g \in (CRG_A \cap CRG_B) \wedge (\Delta_A(g)\Delta_B(g)) < 0\} \wedge \Delta_g \neq 0$$

2, missing genes

$$for\ \{g | g \in (CRG_A \cap CRG_B) \wedge (\Delta_A(g)\Delta_B(g)) < 0 \wedge (\Delta_A + \Delta_B) \neq 0\}$$
$$if\ \frac{1-f_1}{1-f_2} = \frac{-\Delta_B(g)}{\Delta_A(g)} \rightarrow \Delta_g = 0$$
$$for\ \{g | g \in CRG_{A/B}\}$$

if $f_1 = 1$ then $CRG = CRG_B \subseteq CRG_{A/B}$ and *if* $f_2 = 1$ then $CRG = CRG_A \subseteq CRG_{A/B}$

Above all, we believe that $CRG \subseteq CRG_{A/B}$ and most genes in the two gene sets have the same deregulation directions.

End

REFERENCES

1. Endo H, Ikeda K, Urano T, Horie-Inoue K, Inoue S. Terf/TRIM17 stimulates degradation of kinetochore protein ZWINT and regulates cell proliferation. J Biochem. 2012; 151:139–144.
2. Temraz S, Mukherji D, Alameddine R, Shamseddine A. Methods of overcoming treatment resistance in colorectal cancer. Crit Rev Oncol Hematol. 2014; 89:217–230.
3. Gil J, O'Loghlen A. PRC1 complex diversity: where is it taking us?. Trends Cell Biol. 2014; 24:632–641.
4. Morgan MA, Shilatifard A. Chromatin signatures of cancer. Genes Dev. 2015; 29:238–249.
5. Mojardin L, Botet J, Moreno S, Salas M. Chromosome segregation and organization are targets of 5'-Fluorouracil in eukaryotic cells. Cell Cycle. 2015; 14:206–218.
6. Salmela AL, Kallio MJ. Mitosis as an anti-cancer drug target. Chromosoma. 2013; 122:431–449.
7. Seetharam R, Sood A, Goel S. Oxaliplatin: pre-clinical perspectives on the mechanisms of action, response and resistance. Ecancermedicalscience. 2009; 3:153.
8. Liao W, Liu W, Yuan Q, Liu X, Ou Y, He S, Yuan S, Qin L, Chen Q, Nong K, Mei M, Huang J. Silencing of DLGAP5 by siRNA significantly inhibits the proliferation and invasion of hepatocellular carcinoma cells. PLoS One. 2013; 8:e80789.
9. Singel SM, Cornelius C, Batten K, Fasciani G, Wright WE, Lum L, Shay JW. A targeted RNAi screen of the breast cancer genome identifies KIF14 and TLN1 as genes that modulate docetaxel chemosensitivity in triple-negative breast cancer. Clin Cancer Res. 2013; 19:2061–2070.
10. Ishii H, Saitoh M, Sakamoto K, Kondo T, Katoh R, Tanaka S, Motizuki M, Masuyama K, Miyazawa K. Epithelial splicing regulatory proteins 1 (ESRP1) and 2 (ESRP2) suppress cancer cell motility via different mechanisms. J Biol Chem. 2014; 289:27386–27399.
11. Hishiki A, Hara K, Ikegaya Y, Yokoyama H, Shimizu T, Sato M, Hashimoto H. Structure of a Novel DNA-binding Domain of Helicase-like Transcription Factor (HLTF) and Its Functional Implication in DNA Damage Tolerance. J Biol Chem. 2015; 290:13215–13223.

12. Moldovan GL, D'Andrea AD. DNA damage discrimination at stalled replication forks by the Rad5 homologs HLTF and SHPRH. Mol Cell. 2011; 42:141–143.
13. Arai M, Kondoh N, Imazeki N, Hada A, Hatsuse K, Matsubara O, Yamamoto M. The knockdown of endogenous replication factor C4 decreases the growth and enhances the chemosensitivity of hepatocellular carcinoma cells. Liver Int. 2009; 29:55–62.
14. Kaistha BP, Honstein T, Muller V, Bielak S, Sauer M, Kreider R, Fassan M, Scarpa A, Schmees C, Volkmer H, Gress TM, Buchholz M. Key role of dual specificity kinase TTK in proliferation and survival of pancreatic cancer cells. Br J Cancer. 2014; 111:1780–1787.
15. Niittymaki I, Gylfe A, Laine L, Laakso M, Lehtonen HJ, Kondelin J, Tolvanen J, Nousiainen K, Pouwels J, Jarvinen H, Nuorva K, Mecklin JP, Makinen M, Ristimaki A, Orntoft TF, Hautaniemi S, et al. High frequency of TTK mutations in microsatellite-unstable colorectal cancer and evaluation of their effect on spindle assembly checkpoint. Carcinogenesis. 2011; 32:305–311.
16. Huang Y, Sadee W. Membrane transporters and channels in chemoresistance and sensitivity of tumor cells. Cancer Lett. 2006; 239:168–182.
17. Kotian S, Banerjee T, Lockhart A, Huang K, Catalyurek UV, Parvin JD. NUSAP1 influences the DNA damage response by controlling BRCA1 protein levels. Cancer Biol Ther. 2014; 15:533–543.
18. Chen Q, Chen K, Guo G, Li F, Chen C, Wang S, Nalepa G, Huang S, Chen JL. A critical role of CDKN3 in Bcr-Abl-mediated tumorigenesis. PLoS One. 2014; 9:e111611.
19. Vie N, Copois V, Bascoul-Mollevi C, Denis V, Bec N, Robert B, Fraslon C, Conseiller E, Molina F, Larroque C, Martineau P, Del Rio M, Gongora C. Overexpression of phosphoserine aminotransferase PSAT1 stimulates cell growth and increases chemoresistance of colon cancer cells. Mol Cancer. 2008; 7:14.
20. Fan Y, Wang L, Han X, Liu X, Ma H. Rab25 is responsible for phosphoinositide 3-kinase/AKTmediated cisplatin resistance in human epithelial ovarian cancer cells. Mol Med Rep. 2015; 11:2173–2178.
21. Goldenring JR, Nam KT. Rab25 as a tumour suppressor in colon carcinogenesis. Br J Cancer. 2011; 104:33–36.
22. Darido C, Buchert M, Pannequin J, Bastide P, Zalzali H, Mantamadiotis T, Bourgaux JF, Garambois V, Jay P, Blache P, Joubert D, Hollande F. Defective claudin-7 regulation by Tcf-4 and Sox-9 disrupts the polarity and increases the tumorigenicity of colorectal cancer cells. Cancer Res. 2008; 68:4258–4268.
23. Petrakis TG, Vougas K, Gorgoulis VG. Cdc6: a multi-functional molecular switch with critical role in carcinogenesis. Transcription. 2012; 3:124–129.
24. Liu SM, Chen W, Wang J. Distinguishing between cancer cell differentiation and resistance induced by all-trans retinoic acid using transcriptional profiles and functional pathway analysis. Sci Rep. 2014; 4:5577.
25. Can G, Akpinar B, Baran Y, Zhivotovsky B, Olsson M. 5-Fluorouracil signaling through a calcium-calmodulin-dependent pathway is required for p53 activation and apoptosis in colon carcinoma cells. Oncogene. 2013; 32:4529–4538.
26. Zhao Y, Butler EB, Tan M. Targeting cellular metabolism to improve cancer therapeutics. Cell Death Dis. 2013; 4:e532.
27. Kibria G, Hatakeyama H, Harashima H. Cancer multidrug resistance: mechanisms involved and strategies for circumvention using a drug delivery system. Arch Pharm Res. 2014; 37:4–15.
28. Borst P, Evers R, Kool M, Wijnholds J. A family of drug transporters: the multidrug resistance-associated proteins. J Natl Cancer Inst. 2000; 92:1295–1302.
29. Lu CW, Lin SC, Chien CW, Lee CT, Lin BW, Lee JC, Tsai SJ. Overexpression of pyruvate dehydrogenase kinase 3 increases drug resistance and early recurrence in colon cancer. Am J Pathol. 2011; 179:1405–1414.
30. Bonnal S, Vigevani L, Valcarcel J. The spliceosome as a target of novel antitumour drugs. Nat Rev Drug Discov. 2012; 11:847–859.
31. Mitsiades N, Mitsiades CS, Richardson PG, Poulaki V, Tai YT, Chauhan D, Fanourakis G, Gu X, Bailey C, Joseph M, Libermann TA, Schlossman R, Munshi NC, Hideshima T, Anderson KC. The proteasome inhibitor PS-341 potentiates sensitivity of multiple myeloma cells to conventional chemotherapeutic agents: therapeutic applications. Blood. 2003; 101:2377–2380.
32. Martino-Echarri E, Henderson BR, Brocardo MG. Targeting the DNA replication checkpoint by pharmacologic inhibition of Chk1 kinase: a strategy to sensitize APC mutant colon cancer cells to 5-fluorouracil chemotherapy. Oncotarget. 2014; 5:9889–9900.
33. Mandic A, Hansson J, Linder S, Shoshan MC. Cisplatin induces endoplasmic reticulum stress and nucleus-independent apoptotic signaling. J Biol Chem. 2003; 278:9100–9106.
34. Rabik CA, Dolan ME. Molecular mechanisms of resistance and toxicity associated with platinating agents. Cancer Treat Rev. 2007; 33:9–23.
35. Sui X, Kong N, Wang X, Fang Y, Hu X, Xu Y, Chen W, Wang K, Li D, Jin W, Lou F, Zheng Y, Hu H, Gong L, Zhou X, Pan H, et al. JNK confers 5-fluorouracil resistance in p53-deficient and mutant p53-expressing colon cancer cells by inducing survival autophagy. Sci Rep. 2014; 4:4694.

36. Shigeta K, Ishii Y, Hasegawa H, Okabayashi K, Kitagawa Y. Evaluation of 5-fluorouracil metabolic enzymes as predictors of response to adjuvant chemotherapy outcomes in patients with stage II/III colorectal cancer: a decision-curve analysis. World J Surg. 2014; 38:3248–3256.
37. Longley DB, Harkin DP, Johnston PG. 5-fluorouracil: mechanisms of action and clinical strategies. Nat Rev Cancer. 2003; 3:330–338.
38. Carrillo E, Navarro SA, Ramirez A, Garcia MA, Grinan-Lison C, Peran M, Marchal JA. 5-Fluorouracil derivatives: a patent review 2012–2014. Expert Opin Ther Pat. 2015 :1–14.
39. Michelakis ED, Webster L, Mackey JR. Dichloroacetate (DCA) as a potential metabolic-targeting therapy for cancer. Br J Cancer. 2008; 99:989–994.
40. Egler V, Korur S, Failly M, Boulay JL, Imber R, Lino MM, Merlo A. Histone deacetylase inhibition and blockade of the glycolytic pathway synergistically induce glioblastoma cell death. Clin Cancer Res. 2008; 14:3132–3140.
41. Maschek G, Savaraj N, Priebe W, Braunschweiger P, Hamilton K, Tidmarsh GF, De Young LR, Lampidis TJ. 2-deoxy-D-glucose increases the efficacy of adriamycin and paclitaxel in human osteosarcoma and non-small cell lung cancers in vivo. Cancer Res. 2004; 64:31–34.
42. Aft RL, Zhang FW, Gius D. Evaluation of 2-deoxy-D-glucose as a chemotherapeutic agent: mechanism of cell death. Br J Cancer. 2002; 87:805–812.
43. Garnett MJ, Edelman EJ, Heidorn SJ, Greenman CD, Dastur A, Lau KW, Greninger P, Thompson IR, Luo X, Soares J, Liu Q, Iorio F, Surdez D, Chen L, Milano RJ, Bignell GR, et al. Systematic identification of genomic markers of drug sensitivity in cancer cells. Nature. 2012; 483:570–575.
44. Zuco V, Zunino F. Cyclic pifithrin-alpha sensitizes wild type p53 tumor cells to antimicrotubule agent-induced apoptosis. Neoplasia. 2008; 10:587–596.
45. Pietrancosta N, Maina F, Dono R, Moumen A, Garino C, Laras Y, Burlet S, Quelever G, Kraus JL. Novel cyclized Pifithrin-alpha p53 inactivators: synthesis and biological studies. Bioorg Med Chem Lett. 2005; 15:1561–1564.
46. Holohan C, Van Schaeybroeck S, Longley DB, Johnston PG. Cancer drug resistance: an evolving paradigm. Nat Rev Cancer. 2013; 13:714–726.
47. Chou TC. Theoretical basis, experimental design, and computerized simulation of synergism and antagonism in drug combination studies. Pharmacol Rev. 2006; 58:621–681.
48. Peterson JJ, Novick SJ. Nonlinear blending: a useful general concept for the assessment of combination drug synergy. J Recept Signal Transduct Res. 2007; 27(2-3):125–146.
49. Al-Lazikani B, Banerji U, Workman P. Combinatorial drug therapy for cancer in the post-genomic era. Nat Biotechnol. 2012; 30:679–692.

Supplementary Table S1: The consistency scores of the top-ranked 1500 BD and IP_6, IP_{12}, IP_{24} genes detected from HCT1116 cell line

Dataset	Cell line	Drug	Method	IP	Overlapped DEG	Consistent DEG(%)	Binominal *P*-value
E-MEXP-390	HCT116	5-FU	FC	IP_6	510	98.24	<1.11E-16
			AD		987	95.04	<1.11E-16
			FC	IP_{12}	673	98.37	<1.11E-16
			AD		990	93.33	<1.11E-16
			FC	IP_{24}	523	98.28	<1.11E-16
			AD		922	88.61	<1.11E-16
E-MEXP-390	HCT116	L-OHP	FC	IP_6	902	100.00	<1.11E-16
			AD		1178	98.90	<1.11E-16
			FC	IP_{12}	885	100.00	<1.11E-16
			AD		1107	98.84	<1.11E-16
			FC	IP_{24}	672	99.55	<1.11E-16
			AD		1092	93.40	<1.11E-16
E-MEXP-1691	HCT116	5-FU	FC	IP_{24}	853	99.53	<1.11E-16
			AD		1184	97.13	<1.11E-16
E-MEXP-1691	HCT116	SN38	FC	IP_{24}	885	98.76	<1.11E-16
			AD		1171	96.16	<1.11E-16
E-MEXP-1171	HCT116	SN38	FC	IP_6	553	98.69	<1.11E-16
			AD		912	83.23	<1.11E-16
			FC	IP_{12}	445	94.83	<1.11E-16
			AD		933	86.28	<1.11E-16
			FC	IP_{24}	439	92.48	<1.11E-16
			AD		951	90.12	<1.11E-16

Supplementary Table S2: The consistency scores of the top-ranked 3000 (top-ranked 1500) BD genes and IP$_{24}$ genes dectected from other cell line datasets

Dataset	Cell line	Drug	Method	Overlapped DEG[a]	Consistent DEG(%)[b]	Binominal *P*-value[c]
GSE3926	HT29 (colon)	Doxo	FC	1773 (691)	88.16 (91.03)	<4.20E-09 (<4.20E-09)
			AD	1913 (877)	80.55 (83.35)	<4.20E-09 (<4.20E-09)
GSE3926	MCF-7 (breast)	Doxo	FC	1640 (673)	86.10 (93.16)	<4.20E-09 (<4.20E-09)
			AD	1996 (913)	84.62 (86.86)	<4.20E-09 (<4.20E-09)
GSE3926	EPP85-181P (pancreatic)	Doxo	FC	1464 (626)	86.07 (92.49)	<4.20E-09 (<4.20E-09)
			AD	2014 (921)	81.58 (79.80)	<4.20E-09 (<4.20E-09)
GSE3926	EPG85-257P(gastric)	Doxo	FC	1463 (642)	85.92 (91.74)	<4.20E-09 (<4.20E-09)
			AD	2007 (875)	72.35 (74.51)	<4.20E-09 (<4.20E-09)

[a]The number of BD genes overlapped with IP$_{24}$ genes;
[b]The consistency score of BD genes and IP$_{24}$ genes;
[c]The binominal distribution *P*-value.

Supplementary Table S3: The 315 $CRG_{5\text{-}FU/L\text{-}OHP}$ detected from the GSE19860, GSE28702 and E-MEXP-3368 datasets

Geneid	Gene Symbol	Direction[a]
9	NAT1	up
26	ABP1	up
72	ACTG2	up
136	ADORA2B	up
283	ANG	up
339	APOBEC1	up
430	ASCL2	up
688	KLF5	up
701	BUB1B	up
891	CCNB1	up
990	CDC6	up
1015	CDH17	up
1033	CDKN3	up
1113	CHGA	up
1179	CLCA1	up
1363	CPE	up
1365	CLDN3	up
1366	CLDN7	up
1644	DDC	up
1690	COCH	up
1836	SLC26A2	up
2049	EPHB3	up
2150	F2RL1	up
2295	FOXF2	up
2330	FMO5	up
2487	FRZB	up
2494	NR5A2	up
2524	FUT2	up
2525	FUT3	up
2526	FUT4	up
2641	GCG	up
2762	GMDS	up
2921	CXCL3	up
2980	GUCA2A	up
3115	HLA-DPB1	up

(*Continued*)

Geneid	Gene Symbol	Direction[a]
3158	HMGCS2	up
3171	FOXA3	up
3174	HNF4G	up
3207	HOXA11	up
3209	HOXA13	up
3306	HSPA2	up
3574	IL7	up
3613	IMPA2	up
3832	KIF11	up
3957	LGALS2	up
3960	LGALS4	up
4013	VWA5A	up
4128	MAOA	up
4171	MCM2	up
4246	SCGB2A1	up
4306	NR3C2	up
4314	MMP3	up
4499	MT1M	up
4583	MUC2	up
4585	MUC4	up
4602	MYB	up
4640	MYO1A	up
4751	NEK2	up
4982	TNFRSF11B	up
4998	ORC1	up
5105	PCK1	up
5122	PCSK1	up
5205	ATP8B1	up
5284	PIGR	up
5318	PKP2	up
5320	PLA2G2A	up
5325	PLAGL1	up
5357	PLS1	up
5468	PPARG	up
5557	PRIM1	up
5789	PTPRD	up

(*Continued*)

Geneid	Gene Symbol	Direction[a]
5918	RARRES1	up
5984	RFC4	up
5985	RFC5	up
6038	RNASE4	up
6491	STIL	up
6549	SLC9A2	up
6596	HLTF	up
6674	SPAG1	up
6690	SPINK1	up
6717	SRI	up
6920	TCEA3	up
7033	TFF3	up
7098	TLR3	up
7153	TOP2A	up
7263	TST	up
7272	TTK	up
7298	TYMS	up
7368	UGT8	up
7374	UNG	up
7398	USP1	up
7443	VRK1	up
7504	XK	up
7850	IL1R2	up
8618	CADPS	up
8702	B4GALT4	up
8792	TNFRSF11A	up
8857	FCGBP	up
9037	SEMA5A	up
9055	PRC1	up
9134	CCNE2	up
9166	EBAG9	up
9245	GCNT3	up
9314	KLF4	up
9582	APOBEC3B	up
9787	DLGAP5	up
9928	KIF14	up

(*Continued*)

Geneid	Gene Symbol	Direction[a]
9982	FGFBP1	up
10008	KCNE3	up
10020	GNE	up
10103	TSPAN1	up
10112	KIF20A	up
10158	PDZK1IP1	up
10189	ALYREF	up
10223	GPA33	up
10403	NDC80	up
10406	WFDC2	up
10418	SPON1	up
10481	HOXB13	up
10551	AGR2	up
10559	SLC35A1	up
10605	PAIP1	up
10615	SPAG5	up
10753	CAPN9	up
10903	MTMR11	up
11004	KIF2C	up
11005	SPINK5	up
11130	ZWINT	up
23171	GPD1L	up
23321	TRIM2	up
23443	SLC35A3	up
23584	VSIG2	up
25994	HIGD1A	up
26018	LRIG1	up
26060	APPL1	up
26298	EHF	up
26996	GPR160	up
27035	NOX1	up
27075	TSPAN13	up
27283	TINAG	up
27284	SULT1B1	up
27290	SPINK4	up
29091	STXBP6	up

(*Continued*)

Geneid	Gene Symbol	Direction[a]
29968	PSAT1	up
50853	VILL	up
51000	SLC35B3	up
51195	RAPGEFL1	up
51203	NUSAP1	up
51514	DTL	up
51567	TDP2	up
51703	ACSL5	up
51809	GALNT7	up
54474	KRT20	up
54546	RNF186	up
54596	L1TD1	up
54827	FAM55D	up
54836	BSPRY	up
54843	SYTL2	up
54845	ESRP1	up
54847	SIDT1	up
54860	MS4A12	up
54866	PPP1R14D	up
54933	RHBDL2	up
55040	EPN3	up
55179	FAIM	up
55204	GOLPH3L	up
55286	C4orf19	up
55502	HES6	up
55600	ITLN1	up
55635	DEPDC1	up
55711	FAR2	up
55769	ZNF83	up
55808	ST6GALNAC1	up
55930	MYO5C	up
56267	CCBL2	up
56925	LXN	up
56987	BBX	up
56992	KIF15	up
57111	RAB25	up

(*Continued*)

Geneid	Gene Symbol	Direction[a]
57126	CD177	up
57216	VANGL2	up
57405	SPC25	up
57475	PLEKHH1	up
57482	KIAA1211	up
57535	KIAA1324	up
57552	NCEH1	up
57643	ZSWIM5	up
63928	CHP2	up
64073	C19orf33	up
64105	CENPK	up
64417	C5orf28	up
64426	SUDS3	up
64922	LRRC19	up
79083	MLPH	up
79170	PRR15L	up
79682	MLF1IP	up
79730	NSUN7	up
79733	E2F8	up
79782	LRRC31	up
79789	CLMN	up
80117	ARL14	up
80150	ASRGL1	up
80157	CWH43	up
80736	SLC44A4	up
81575	APOLD1	up
81618	ITM2C	up
83661	MS4A8B	up
83699	SH3BGRL2	up
84057	MND1	up
84152	PPP1R1B	up
84302	TMEM246	up
84419	C15orf48	up
84708	LNX1	up
84842	HPDL	up
85315	PAQR8	up
90333	ZNF468	up

(*Continued*)

Geneid	Gene Symbol	Direction[a]
91392	ZNF502	up
112609	MRAP2	up
113802	HENMT1	up
114907	FBXO32	up
116832	RPL39L	up
120400	FAM55A	up
124975	GGT6	up
131076	CCDC58	up
131177	FAM3D	up
138065	RNF183	up
139886	SPIN4	up
145376	PPP1R36	up
148170	CDC42EP5	up
148418	SAMD13	up
149175	MANEAL	up
150209	AIFM3	up
151246	SGOL2	up
151473	SLC16A14	up
151827	LRRC34	up
152100	CMC1	up
155465	AGR3	up
171546	SPTSSA	up
192134	B3GNT6	up
200958	MUC20	up
220963	SLC16A9	up
221061	FAM171A1	up
221443	C6orf130	up
222171	PRR15	up
253012	HEPACAM2	up
255738	PCSK9	up
282809	POC1B	up
285704	RGMB	up
340277	FAM221A	up
401546	C9orf152	up
653808	ZG16	up
100133941	CD24	up

(*Continued*)

Geneid	Gene Symbol	Direction[a]
341	APOC1	down
684	BST2	down
712	C1QA	down
952	CD38	down
978	CDA	down
1109	AKR1C4	down
1118	CHIT1	down
1244	ABCC2	down
1299	COL9A3	down
1308	COL17A1	down
1687	DFNA5	down
1847	DUSP5	down
1959	EGR2	down
2213	FCGR2B	down
3310	HSPA6	down
3315	HSPB1	down
3397	ID1	down
3569	IL6	down
3911	LAMA5	down
4283	CXCL9	down
4318	MMP9	down
4547	MTTP	down
5010	CLDN11	down
5054	SERPINE1	down
5360	PLTP	down
5621	PRNP	down
6347	CCL2	down
6352	CCL5	down
6362	CCL18	down
6387	CXCL12	down
6515	SLC2A3	down
6581	SLC22A3	down
7134	TNNC1	down
7305	TYROBP	down
7345	UCHL1	down
8529	CYP4F2	down
9353	SLIT2	down

(*Continued*)

Geneid	Gene Symbol	Direction[a]
9945	GFPT2	down
10232	MSLN	down
10457	GPNMB	down
10544	PROCR	down
10673	TNFSF13B	down
10974	C10orf116	down
11156	PTP4A3	down
11326	VSIG4	down
23650	TRIM29	down
26049	FAM169A	down
27345	KCNMB4	down
50861	STMN3	down
51330	TNFRSF12A	down
51365	PLA1A	down
51816	CECR1	down
54749	EPDR1	down
57834	CYP4F11	down
60681	FKBP10	down
81035	COLEC12	down
84647	PLA2G12B	down
90293	KLHL13	down
91373	UAP1L1	down
118471	PRAP1	down
140453	MUC17	down
147920	IGFL2	down
151887	CCDC80	down

Note: [a]The direction of DEGs computed by the RankProduct method: Each gene was defined as up-regulated (or down-regulated) in non– responders compared with responders if the FDR <0.2 in one dataset and with *P*-value <0.05 in another.

Supplementary Table S4: The 131 $CRG_{5\text{-}FU}$ detected from the $CRG_{5\text{-}FU/L\text{-}OHP}$ and GSE52735 dataset

Geneid	Gene Symbol	Direction[a]
9	NAT1	up
136	ADORA2B	up
339	APOBEC1	up
430	ASCL2	up
688	KLF5	up
701	BUB1B	up
891	CCNB1	up
990	CDC6	up
1033	CDKN3	up
1363	CPE	up
1365	CLDN3	up
1366	CLDN7	up
1644	DDC	up
2049	EPHB3	up
2150	F2RL1	up
2487	FRZB	up
2525	FUT3	up
2526	FUT4	up
2762	GMDS	up
2921	CXCL3	up
3171	FOXA3	up
3832	KIF11	up
3960	LGALS4	up
4013	VWA5A	up
4171	MCM2	up
4246	SCGB2A1	up
4306	NR3C2	up
4585	MUC4	up
4751	NEK2	up
4982	TNFRSF11B	up
5122	PCSK1	up
5318	PKP2	up
5357	PLS1	up
5468	PPARG	up
5557	PRIM1	up
5789	PTPRD	up

(*Continued*)

Geneid	Gene Symbol	Direction[a]
5918	RARRES1	up
5984	RFC4	up
5985	RFC5	up
6491	STIL	up
6596	HLTF	up
6674	SPAG1	up
6690	SPINK1	up
7033	TFF3	up
7272	TTK	up
7368	UGT8	up
7443	VRK1	up
7504	XK	up
7850	IL1R2	up
8792	TNFRSF11A	up
9055	PRC1	up
9314	KLF4	up
9582	APOBEC3B	up
9787	DLGAP5	up
9928	KIF14	up
9982	FGFBP1	up
10020	GNE	up
10223	GPA33	up
10403	NDC80	up
10418	SPON1	up
10481	HOXB13	up
10551	AGR2	up
10753	CAPN9	up
11004	KIF2C	up
11130	ZWINT	up
23443	SLC35A3	up
26298	EHF	up
26996	GPR160	up
27075	TSPAN13	up
29091	STXBP6	up
29968	PSAT1	up
51514	DTL	up
51809	GALNT7	up

(*Continued*)

Geneid	Gene Symbol	Direction[a]
54596	L1TD1	up
54836	BSPRY	up
54845	ESRP1	up
55286	C4orf19	up
55502	HES6	up
55635	DEPDC1	up
55930	MYO5C	up
56992	KIF15	up
57111	RAB25	up
57405	SPC25	up
57475	PLEKHH1	up
57552	NCEH1	up
64073	C19orf33	up
64105	CENPK	up
79170	PRR15L	up
79733	E2F8	up
79782	LRRC31	up
80150	ASRGL1	up
81575	APOLD1	up
81618	ITM2C	up
84057	MND1	up
84302	TMEM246	up
84419	C15orf48	up
85315	PAQR8	up
124975	GGT6	up
138065	RNF183	up
139886	SPIN4	up
148170	CDC42EP5	up
148418	SAMD13	up
149175	MANEAL	up
150209	AIFM3	up
151827	LRRC34	up
152100	CMC1	up
192134	B3GNT6	up
222171	PRR15	up
253012	HEPACAM2	up
255738	PCSK9	up

(*Continued*)

Geneid	Gene Symbol	Direction[a]
282809	POC1B	up
285704	RGMB	up
340277	FAM221A	up
401546	C9orf152	up
100133941	CD24	up
341	APOC1	down
978	CDA	down
1118	CHIT1	down
1959	EGR2	down
3310	HSPA6	down
3315	HSPB1	down
4547	MTTP	down
5010	CLDN11	down
6347	CCL2	down
7134	TNNC1	down
8529	CYP4F2	down
9353	SLIT2	down
10457	GPNMB	down
10974	C10orf116	down
84647	PLA2G12B	down
151887	CCDC80	down

Note: [a]The direction of DEGs computed by the RankProduct method: Each gene was defined as up-regulated (or down-regulated) in non–responders compared with responders in both $CRG_{5\text{-FU/L-OHP}}$ and the GSE52735 dataset(FDR<0.2).

Supplementary Table S5: The consistency scores of the top-ranked 300(1500) BD genes or ID genes of 5-FU and L-OHP

Dataset	Cell line	Gene set	Method	Overlapped DEG[a]	Consistent DEG(%)[b]	Binominal *P*-value
E-MEXP-390	HCT116 (colon)	BD	**FC**	**1312(575)**	**97.13(97.22)**	**<5.55E-16(<5.55E-16)**
			**AD**	**1980(888)**	**80.51(81.10)**	**<5.55E-16(<5.55E-16)**
		ID_6	FC	681(281)	61.38(65.12)	1.55E-09(2.23E-07)
			AD	1783(832)	48.63(44.11)	>1.00E-01(>1.00E-0.1)
		ID_{12}	**FC**	**784(282)**	**79.72(82.27)**	**<5.55E-16(<5.55E-16)**
			**AD**	**1834(807)**	**70.61(73.73)**	**<5.55E-16(<5.55E-16)**
		ID_{24}	**FC**	**580(196)**	**66.55(70.92)**	**5.55E-16(2.17E-09)**
			**AD**	**1921(917)**	**91.10(94.44)**	**<5.55E-16(<5.55E-16)**

[a]The number of BD genes (ID genes) of 5-FU overlapped with BD genes (ID genes) of L-OHP;
[b]The consistency score of BD genes (ID genes) of 5-FU and BD genes (ID genes) of L-OHP;

Supplementary Table S6: The consistency scores of CRG_{5-FU} and the top-ranked 300(1500) BD or ID genes

Dataset	Cell line	Gene set	Method	Overlapped DEG[a]	Consistent DEG(%)	Binominal *P*-value
E-MEXP-390	HCT116	BD	FC	26(18)	50.00 (55.56)	>1.00E-01 (>1.00E-01)
			**AD**	38(22)	**65.79 (68.18)**	**3.53E-02 (6.69E-02)**
		ID_6	FC	32(17)	46.88 (52.94)	>1.00E-01 (>1.00E-01)
			AD	37(21)	40.54 (38.10)	>1.00E-01 (>1.00E-01)
		ID_{12}	**FC**	38(23)	**86.84 (91.30)**	**2.13E-06 (3.30E-05)**
			**AD**	52(31)	**80.77 (87.10)**	**4.53E-06 (1.70E-05)**
		ID_{24}	FC	30(16)	66.67 (62.60)	4.94E-02 (>1.00E-01)
			**AD**	41(25)	**75.61 (72.00)**	**7.25E-04 (2.16E-02)**

Supplementary Table S7: The consistency scores of $CRG_{5\text{-}FU/L\text{-}OHP}$ and the top-ranked 1500 BD or ID genes

Data source	Drug	Cell line	Gene set	Method	Overlapped DEG[b]	Consistent DEG(%)	Binominal *P*-value
E-MEXP-390	5-FU/L-OHP	HCT116	BD_{two}	FC	24	45.83	>1.00E-0.1
				AD	25	60.00	>1.00E-0.1
			$ID_{two\text{-}6}$	FC	5	40.00	>1.00E-0.1
				AD	12	75.00	7.30E-02
			$ID_{two\text{-}12}$	FC	6	50.00	>1.00E-0.1
				AD	18	50.00	>1.00E-0.1
			$ID_{two\text{-}24}$	FC	3	100.00	>1.00E-0.1
				**AD**	**23**	**82.61**	**1.30E-03**
E-MEXP-390	5-FU	HCT116	BD	FC	45	53.33	>1.00E-0.1
				AD	44	68.18	1.13E-02
			ID_6	FC	46	54.35	>1.00E-0.1
				AD	40	50.00	>1.00E-0.1
			ID_{12}	**FC**	**46**	**82.61**	**4.62E-06**
				**AD**	**51**	**78.43**	**2.85E-05**
			ID_{24}	FC	47	53.19	>1.00E-0.1
				**AD**	**46**	**73.91**	**8.21E-04**
E-MEXP-390	L-OHP	HCT116	BD	FC	59	47.46	>1.00E-0.1
				AD	43	55.81	>1.00E-0.1
			ID_6	FC	42	47.62	>1.00E-0.1
				AD	38	57.89	>1.00E-0.1
			ID_{12}	FC	58	17.24	>1.00E-0.1
				AD	58	25.86	>1.00E-0.1
			ID_{24}	**FC**	**42**	**80.95**	**3.44E-05**
				**AD**	**42**	**88.10**	**2.22E-07**
GSE10405	L-OHP	DLD1	BD	FC	8	87.50	3.52E-02
				AD	8	87.50	3.52E-02
		HT29		FC	8	37.50	>1.00E-0.1
				AD	8	50.00	>1.00E-0.1
		LS513		FC	8	62.50	>1.00E-0.1
				AD	8	62.50	>1.00E-0.1
		Lovo		FC	8	50.00	>1.00E-0.1
				AD	6	50.00	>1.00E-0.1

[a]The number of candidate drug resistance genes overlapped with $CRG_{5\text{-}FU/L\text{-}OHP}$;
[b]The consistency score of candidate drug resistance genes and $CRG_{5\text{-}FU/L\text{-}OHP}$.

Supplementary Table S8: The average expression levels of DEGs exclusively detected by FC or AD and the corresponding consistency scores with $CRG_{5\text{-}FU/L\text{-}OHP}$ or $ID_{clinical}$ genes

Gene set	Cell line sample	Method	Average expression	Overlapped DEG	Consistent DEG(%)	Binominal *P*-value
$ID_{two\text{-}24}$[a]	parental cell line treatd with 5-FU for 24h	FC	212.93	7	57.14	>1.00E-0.1
	resistant cell line treatd with 5-FU for for 24h		238.03			
	parental cell line treatd with 5-FU for 24h	**AD**	**1992.72**	**35**	**82.86**	**5.84E-05**
	resistant cell line treatd with 5-FU for for 24h		**2322.72**			
	parental cell line treatd with L-OHP for 24h	FC	189.94	7	57.14	>1.00E-0.1
	resistant cell line treatd with L-OHP for for 24h		215.84			
	parental cell line treatd with L-OHP for 24h	**AD**	**1898.34**	**35**	**82.86**	**5.84E-05**
	resistant cell line treatd with L-OHP for for 24h		**2315.57**			
BD	parental cell line	FC	124.36	40	35.00	>1.00E-0.1
	5-FU resistant cell line		109.79			
	parental cell line	**AD**	**2187.87**	**36**	**66.67**	**3.26E-02**
	5-FU resistant cell line		**2178.89**			
ID_{24}	parental cell line treatd with 5-FU for 24h	FC	132.29	52	44.23	>1.00E-0.1
	resistant cell line treatd with 5-FU for for 24h		133.43			
	parental cell line treatd with 5-FU for 24h	**AD**	**1859.91**	**52**	**69.23**	**3.89E-03**
	resistant cell line treatd with 5-FU for for 24h		**1979.44**			
$ID_{two\text{-}24}$[b]	parental cell line treatd with 5-FU for 24h	FC	129.16	54	57.41	>1.00E-0.1
	resistant cell line treatd with 5-FU for for 24h		119.5			
	parental cell line treatd with 5-FU for 24h	**AD**	**2205.26**	**131**	**73.28**	**4.70E-08**
	resistant cell line treatd with 5-FU for for 24h		**2537.18**			
	parental cell line treatd with L-OHP for 24h	FC	126.76	54	57.41	>1.00E-0.1
	resistant cell line treatd with L-OHP for for 24h		118.39			
	parental cell line treatd with L-OHP for 24h	**AD**	**2114.29**	**131**	**73.28**	**4.70E-08**
	resistant cell line treatd with L-OHP for for 24h		**2457.48**			

Abbreviations:
[a]$ID_{two\text{-}24}$ genes compared with $CRG_{5\text{-}FU/L\text{-}OHP}$;
[b]$ID_{two\text{-}24}$ genes compared with $ID_{clinical}$ genes.

Supplementary Table S9: 70 genes of 5-FU resistance and 65 genes of L-OHP resistance

Drug	Gene Symbol	FC value	AD value	Direction[a]
5-FU	BUB1B	1.02	439.79	up
	B4GALT4	1.04	76.99	up
	CCDC58	1.02	177.84	up
	CCNB1	1.05	443.07	up
	CD24	1.03	155.64	up
	CENPK	1.03	76.99	up
	CLDN3	1.04	77.80	up
	CMC1	1.04	272.91	up
	DEPDC1	1.02	92.57	up
	DLGAP5	1.02	369.33	up
	EBAG9	1.03	190.08	up
	ESRP1	1.02	280.04	up
	FAM171A1	1.03	281.89	up
	GNE	1.04	54.55	up
	GOLPH3L	1.02	170.37	up
	GPR160	1.04	128.67	up
	HES6	1.02	199.86	up
	HLTF	1.01	271.28	up
	HOXA13	1.03	25.51	up
	IMPA2	1.09	576.44	up
	KIAA1211	1.06	69.34	up
	KIF14	1.05	360.68	up
	KIF20A	1.02	168.29	up
	MANEAL	1.02	79.43	up
	MAOA	1.04	85.02	up
	MCM2	1.01	213.23	up
	MND1	1.03	116.63	up
	MYO5C	1.02	221.51	up
	NDC80	1.06	519.47	up
	NR3C2	1.04	15.83	up
	NR5A2	1.06	12.53	up
	PAIP1	1.04	336.38	up
	PLAGL1	1.04	111.00	up
	PLEKHH1	1.08	138.95	up
	PLS1	1.01	152.86	up
	PPARG	1.05	143.69	up

(Continued)

Drug	Gene Symbol	FC value	AD value	Direction[a]
5-FU	PRC1	1.02	563.74	up
	PSAT1	1.01	244.31	up
	RAB25	1.01	101.14	up
	RFC4	1.01	267.77	up
	RNASE4	1.08	89.26	up
	SGOL2	1.03	121.68	up
	SLC16A14	1.04	51.90	up
	SLC35A1	1.03	234.17	up
	SLC35A3	1.04	68.34	up
	SPTSSA	1.02	101.42	up
	SRI	1.06	180.90	up
	STXBP6	1.08	14.70	up
	SUDS3	1.05	203.54	up
	SYTL2	1.03	100.64	up
	TDP2	1.02	210.86	up
	TSPAN13	1.02	365.85	up
	TST	1.01	108.90	up
	TTK	1.02	241.92	up
	TYMS	1.04	648.87	up
	UNG	1.03	415.74	up
	VWA5A	1.04	83.72	up
	XK	1.05	58.94	up
	ZNF468	1.06	133.06	up
	ZWINT	1.02	903.01	up
	ABCC2	1.03	96.44	down
	DUSP5	1.01	231.34	down
	LAMA5	1.02	171.80	down
	SERPINE1	1.05	57.56	down
	PLTP	1.04	27.92	down
	TNNC1	1.11	50.50	down
	UCHL1	1.05	25.91	down
	CYP4F2	1.04	28.27	down
	PROCR	1.02	195.37	down
	VSIG4	1.06	40.57	down
L-OHP	ALYREF	1.05	1351.19	up
	ANG	1.04	32.78	up
	BUB1B	1.04	646.75	up

(*Continued*)

Drug	Gene Symbol	FC value	AD value	Direction[a]
L-OHP	CCDC58	1.02	256.12	up
	CCNB1	1.02	221.64	up
	CCNE2	1.13	270.36	up
	CD24	1.02	135.36	up
	CDC6	1.07	427.31	up
	CDKN3	1.04	550.51	up
	CENPK	1.07	103.74	up
	CENPU	1.06	139.69	up
	CHGA	1.03	13.48	up
	CLDN3	1.04	60.97	up
	CLDN7	1.03	499.85	up
	CMC1	1.04	305.48	up
	DLGAP5	1.02	205.55	up
	DTL	1.07	418.38	up
	E2F8	1.06	165.21	up
	EBAG9	1.02	166.11	up
	ESRP1	1.02	322.77	up
	F2RL1	1.02	321.75	up
	FAIM	1.04	71.36	up
	HES6	1.02	177.93	up
	HLTF	1.02	358.03	up
	HPDL	1.05	20.85	up
	IMPA2	1.08	327.21	up
	ITM2C	1.04	493.52	up
	KIF11	1.05	211.65	up
	KIF14	1.03	128.62	up
	KIF20A	1.02	140.89	up
	KIF2C	1.03	350.41	up
	LGALS2	1.04	9.27	up
	MANEAL	1.05	130.07	up
	MCM2	1.05	1276.48	up
	MND1	1.08	252.19	up
	MYO5C	1.02	187.17	up
	NDC80	1.04	246.25	up
	NEK2	1.03	139.92	up
	NUSAP1	1.04	564.83	up
	ORC1	1.06	103.89	up

(*Continued*)

Drug	Gene Symbol	FC value	AD value	Direction[a]
L-OHP	PAIP1	1.03	254.04	up
	PCSK9	1.04	128.89	up
	PDZK1IP1	1.03	12.34	up
	PLS1	1.01	115.53	up
	PRC1	1.04	1029.61	up
	PRIM1	1.05	926.98	up
	PSAT1	1.03	511.77	up
	RAB25	1.03	506.02	up
	RFC4	1.05	855.13	up
	RFC5	1.05	360.68	up
	SLC9A2	1.04	10.65	up
	SPAG5	1.03	139.38	up
	SPC25	1.08	255.39	up
	SPIN4	1.05	260.68	up
	STIL	1.03	159.80	up
	STXBP6	1.05	9.52	up
	TTK	1.03	296.06	up
	TYMS	1.03	278.62	up
	UNG	1.06	805.10	up
	USP1	1.05	316.55	up
	VRK1	1.03	245.80	up
	VSIG2	1.04	25.49	up
	ZG16	1.05	26.82	up
	ZWINT	1.04	1235.85	up
	IGFL2	1.06	111.20	down

[a]The direction of DEGs: Each gene was defined as up-regulated (or downregulated) in resistant cells compared with parental cells if the value of FC or AD was larger (or smaller) than zero.

Supplementary Table S10: Summaries of the top 20 ID_{24} genes ranked by AD method for 5-FU and L-OHP, respectively. All of the genes were up-regulated in the resistant cells compared with the sensitive cells

Drug	Gene Symbol	Summary
5-FU	ZWINT	ZW10 interacting kinetochore protein; The encoded protein is involved in kinetochore function and overexpression of ZWINT stimulates cell growth [1].
	TYMS	thymidylate synthetase; It is a target for 5-FU.High expression of this gene activates 5-FU resistance [2].
	IMPA2	inositol(myo)-1(or 4)-monophosphatase 2;The encoded protein catalyzes the dephosphoylration of inositol monophosphate.
	PRC1	protein regulator of cytokinesis 1;This gene encodes a protein that is involved in cytokinesis and acts as both tumor suppressors and oncogenes [3].
	NDC80	NDC80 kinetochore complex component;This protein is required for proper chromosome segregation,which might be one of the mechanisms of 5-FU action [4, 5].
	CCNB1	cyclin B1; The protein encoded by this gene is a regulatory protein involved in mitosis,which could be an anti-cancer drug target [6].
	BUB1B	BUB1 mitotic checkpoint serine/threonine kinase B;
	UNG	uracil-DNA glycosylase; UNG initiated base excision repair, which could stimulate the development of be related to L-OHP resistance [7].
	DLGAP5	discs, large (Drosophila) homolog-associated protein 5; Up-regulation of DLGAP5 contributes to hepatocellular carcinoma cells tumorigenesis by promoting cell proliferation [8].
	TSPAN13	tetraspanin 13; The proteins mediate signal transduction events that play a role in the regulation of cell development, activation, growth and motility.
	KIF14	kinesin family member 14;KIF14 knockdown clearly enhanced chemosensitivity to docetaxel in breast cancer and this gene played a role in response to cytotoxic chemotherapy [9].
	PAIP1	poly(A) binding protein interacting protein 1;
	FAM171A1	family with sequence similarity 171, member A1;
	ESRP1	epithelial splicing regulatory protein 1;ESRP1 is re-expressed in the lymph nodes, where carcinoma cells metastasize and colonize [10].
	CMC1	C-x(9)-C motif containing 1;
	HLTF	helicase-like transcription factor; The encoded protein plays a pivotal role in the template-switching pathway of DNA damage tolerance [11, 12].
	RFC4	replication factor C (activator 1) 4, 37kDa; This inhibition of RFC4 expression correlated with a decrease in cellular proliferation, increased levels of apoptosis and a sensitizing of the cells to the DNA-damaging chemotherapeutic agents [13].
	PSAT1	phosphoserine aminotransferase 1;
	TTK	TTK protein kinase; The encoded protein is associated with cell proliferation and essential for chromosome alignment at the centromere during mitosis [14, 15].
	SLC35A1	solute carrier family 35 (CMP-sialic acid transporter), member A1;SLC35A1 is a member of solute carriers (SLCs) and its overexpression can activate the process of absorption and transport of cell inhibitors [16].

(*Continued*)

Drug	Gene Symbol	Summary
L-OHP	ALYREF	Aly/REF export factor;
	MCM2	minichromosome maintenance complex component 2;The protein encoded by this gene is involved in replication and promotes tumor cell proliferation [14].
	ZWINT	ZW10 interacting kinetochore protein; The encoded protein is involved in kinetochore function and overexpression of ZWINT stimulates cell growth [1].
	PRC1	protein regulator of cytokinesis 1;This gene encodes a protein that is involved in cytokinesis.
	PRIM1	primase, DNA, polypeptide 1 (49kDa);
	RFC4	replication factor C (activator 1) 4, 37kDa; This inhibition of RFC4 expression correlated with a decrease in cellular proliferation, increased levels of apoptosis and a sensitizing of the cells to the DNA-damaging chemotherapeutic agents [13].
	UNG	uracil-DNA glycosylase; UNG initiated base excision repair, which could stimulate the development of be related to L-OHP resistance [7].
	BUB1B	BUB1 mitotic checkpoint serine/threonine kinase B;
	NUSAP1	nucleolar and spindle associated protein 1;NUSAP1 influences the DNA damage response by controlling BRCA1 protein levels [17].
	CDKN3	cyclin-dependent kinase inhibitor 3;The gene plays a keyrole in regulating cell division and tumorigenesis [18].
	PSAT1	phosphoserine aminotransferase 1;Overexpression of PSAT1 stimulates cell growth and increases chemoresistance of colon cancer cells to L-OHP [19].
	RAB25	member RAS oncogene family;RAB2is a tumour suppressor in colon carcinogenesis and shows an effect comparable with blocking the PI3K/AKT pathway and contributes to cisplatin resistance in human epithelial ovarian [20, 21].
	CLDN7	claudin 7;Defective Claudin-7 increases the tumorigenicity ofcolorectal cancer cells [22].
	ITM2C	integral membrane protein 2C;
	CDC6	cell division cycle 6;The encoded protein plays a critical role in carcinogenesis [23].
	DTL	denticleless E3 ubiquitin protein ligase homolog (Drosophila);
	RFC5	replication factor C (activator 1) 5, 36.5kDa; The encoded protein is related to mismatch repair [24].
	HLTF	helicase-like transcription factor; The encoded protein plays a pivotal role in the template-switching pathway of DNA damage tolerance [11, 12].
	KIF2C	kinesin family member 2C;
	IMPA2	inositol(myo)-1(or 4)-monophosphatase 2;The encoded protein catalyzes the dephosphoylration of inositol monophosphate.

Supplementary Table S11: The enriched pathways of the top-ranked 3000 DEGs. *p*(<0.05) was adjusted by Benjamini and Hochberg(FDR<0.1)

Drug	Method	KEGG Pathway	*P*-value	Reference
5-FU	FC	**Steroid biosynthesis**	**2.40E-06**	
		Arachidonic acid metabolism	1.69E-03	
		Cytokine-cytokine receptor interaction	2.44E-07	
		Neuroactive ligand-receptor interaction	9.00E-06	
		**p53 signaling pathway**	**8.59E-12**	[25]
		Complement and coagulation cascades	8.61E-05	
	AD	Glycolysis / Gluconeogenesis	1.02E-04	[26]
		**Steroid biosynthesis**	**3.57E-03**	
		Oxidative phosphorylation	3.69E-07	
		Glutathione metabolism	2.81E-03	[27, 28]
		N-Glycan biosynthesis	3.62E-03	
		Amino sugar and nucleotide sugar metabolism	7.28E-03	
		Pyruvate metabolism	1.94E-04	[26, 29]
		Propanoate metabolism	3.70E-03	
		Carbon metabolism	5.48E-03	[26]
		Ribosome biogenesis in eukaryotes	5.44E-07	
		Ribosome	0.00E+00	
		RNA transport	9.21E-06	
		mRNA surveillance pathway	6.79E-05	
		Basal transcription factors	6.53E-04	
		Spliceosome	1.92E-11	[30]
		Proteasome	2.03E-07	[31]
		Protein export	4.52E-05	
		Cell cycle	4.12E-05	[32]
		Protein processing in endoplasmic reticulum	2.04E-08	[33] [34]
		**p53 signaling pathway**	**<1.11E-16**	[25, 35]
		Collecting duct acid secretion	6.39E-04	
L-OHP	FC	Steroid biosynthesis	1.36E-04	
		**Pyrimidine metabolism**	**1.81E-05**	[36]
		Biosynthesis of unsaturated fatty acids	9.42E-04	
		Fatty acid metabolism	2.66E-03	[26]
		**DNA replication**	**9.10E-15**	[32]
		**Base excision repair**	**1.68E-05**	[7]
		**Nucleotide excision repair**	**1.23E-03**	[7]
		**Mismatch repair**	**8.39E-06**	[7, 37]
		Homologous recombination	1.03E-04	

(*Continued*)

Drug	Method	KEGG Pathway	*P*-value	Reference
L-OHP	FC	Fanconi anemia pathway	2.19E-05	
		**Cell cycle**	**8.48E-11**	[32]
		Cytokine-cytokine receptor interaction	2.49E-06	
		Neuroactive ligand-receptor interaction	8.96E-08	
		Olfactory transduction	7.77E-05	
	AD	Glycolysis / Gluconeogenesis	8.07E-06	[26]
		Citrate cycle (TCA cycle)	2.85E-05	[26]
		Pentose phosphate pathway	5.09E-04	
		Fatty acid degradation	1.38E-02	
		Oxidative phosphorylation	1.48E-14	
		**Pyrimidine metabolism**	**9.42E-05**	[36]
		Cysteine and methionine metabolism	2.49E-03	
		Arginine and proline metabolism	3.24E-03	
		Glutathione metabolism	9.56E-04	[27, 28]
		Pyruvate metabolism	2.46E-06	[26, 29]
		Propanoate metabolism	5.43E-04	
		Aminoacyl-tRNA biosynthesis	7.41E-03	
		Carbon metabolism	1.40E-09	[26]
		2-Oxocarboxylic acid metabolism	4.41E-03	
		Biosynthesis of amino acids	6.95E-07	
		Ribosome biogenesis in eukaryotes	2.15E-05	
		Ribosome	<1.11E-16	
		RNA transport	6.33E-11	
		mRNA surveillance pathway	2.96E-03	
		**DNA replication**	**1.02E-10**	[32, 37]
		Spliceosome	9.77E-15	[30]
		Proteasome	1.49E-11	[31]
		Protein export	5.00E-05	
		**Base excision repair**	**1.29E-06**	[7]
		**Nucleotide excision repair**	**4.21E-06**	[7]
		**Mismatch repair**	**2.44E-05**	[7, 37]
		**Cell cycle**	**3.81E-12**	[32]
		Oocyte meiosis	6.53E-03	
		Protein processing in endoplasmic reticulum	1.61E-05	[33, 34]
		Phagosome	1.15E-02	
		p53 signaling pathway	6.75E-06	[25, 35]

Note: Bold parts are common pathways that DEGs ranked by FC or AD significantly enriched in.

Supplementary Table S12: Several inhibitors which have been reported to target the corresponding pathways

KEGG Pathway	Inhibitor
Pyrimidine metabolism	GW776;5-chloro-2,4-dihydroxypyridine (CDHP; gimestat);potassium oxonate (OXO; otastat) [38]
Pyruvate metabolism	Dichloroacetate (DCA) [39]
Glycolysis / Gluconeogenesis	2-deoxy-d-glucose [40–42]
Cell cycle	AZD7762; Roscovitine;CGP-60474;CGP-082996;681640;PD-0332991 [43]
DNA replication	AZD7762;MK-8776 (SCH900776); IC-83 (LY2603618) [32];Camptothecin [43]
DNA Repair	AZD-2281;ABT-888;KU-55933;NU-7441 [43]
p53 signaling pathway	Cyclic Pifithrin-α hydrobromide (ab144327); cyclic dehydrated 6b analogue [44, 45]
Proteasome	bortezomib [46]

Supplementary Table S13: The consistency scores of $ID_{clinical}$ genes and $CRG_{5\text{-}FU/L\text{-}OHP}$ ($CRG_{5\text{-}FU}$) or the top-ranked 3000(top-ranked 1500) ID genes

Drug	Gene set	Method	Overlapped DEG[b]	Consistent DEG(%)	Binominal *P*-value
5-FU/L-OHP	$CRG_{5\text{-}FU/L\text{-}OHP}$	**RankProduct**	**161**	**83.85**	**<1.11E-16**
5-FU	$CRG_{5\text{-}FU}$	**RankProduct**	**78**	**88.46**	**<1.11E-16**
5-FU/L-OHP	$ID_{two\text{-}6}$	FC	49 (27)	36.73 (33.33)	>1.00E-0.1 (>1.00E-0.1)
		AD	95 (43)	56.84 (65.12)	>1.00E-0.1 (3.30E-02)
	$ID_{two\text{-}12}$	FC	87 (40)	47.13 (37.50)	>1.00E-0.1 (>1.00E-0.1)
		AD	138 (63)	44.20 (39.68)	>1.00E-0.1 (>1.00E-0.1)
	$ID_{two\text{-}24}$	FC	63 (27)	60.32 (51.58)	6.50E-02 (>1.00E-0.1)
		**AD**	**140 (59)**	**73.57 (77.97)**	**1.09E-08 (9.58E-06)**
5-FU	ID_{6}	FC	372 (214)	54.84 (55.14)	3.47E-02 (7.55E-02)
		**AD**	**338 (164)**	**57.69 (59.15)**	**2.73E-03 (1.16E-02)**
	ID_{12}	**FC**	**412 (250)**	**58.50 (58.40)**	**3.29E-04 (4.69E-03)**
		**AD**	**372 (199)**	**57.53 (58.79)**	**2.14E-03 (7.87E-03)**
	ID_{24}	**FC**	**395 (234)**	**64.30 (61.97)**	**7.02E-09 (1.52E-04)**
		**AD**	**322 (159)**	**67.08 (67.30)**	**4.31E-10 (7.69E-06)**
L-OHP	ID_{6}	FC	300 (174)	40.33 (37.36)	>1.00E-0.1 (>1.00E-0.1)
		AD	301 (140)	49.83 (51.43)	>1.00E-0.1 (>1.00E-0.1)
	ID_{12}	FC	386 (227)	31.35 (21.59)	>1.00E-0.1 (>1.00E-0.1)
		AD	339 (209)	34.22 (28.23)	>1.00E-0.1 (>1.00E-0.1)
	ID_{24}	**FC**	**312 (172)**	**62.50 (65.70)**	**5.91E-06 (2.33E-05)**
		**AD**	**282 (141)**	**68.44 (75.18)**	**2.76E-10 (8.47E-10)**
